# Survival and Radiotherapy-Related Adverse Events in Patients Receiving Radiotherapy and Concurrent Metformin: A Systematic Review and Meta-Analysis of Randomised Controlled Trials and Cohort Studies

**DOI:** 10.3390/ph18091390

**Published:** 2025-09-17

**Authors:** Wan-Chuen Liao, Hala Shokr, Corinne Faivre-Finn, Clare Dempsey, Kaye Janine Williams, Li-Chia Chen

**Affiliations:** 1 Division of Pharmacy and Optometry, School of Health Sciences, Faculty of Biology, Medicine and Health, The University of Manchester, Manchester M13 9PT, UK; d08450001@ntu.edu.tw (W.-C.L.); kaye.williams@manchester.ac.uk (K.J.W.); li-chia.chen@manchester.ac.uk (L.-C.C.); 2 School of Dentistry, College of Medicine, National Taiwan University, Taipei 106319, Taiwan; 3 Division of Cancer Sciences, Faculty of Biology, Medicine and Health, The University of Manchester, Manchester M13 9PT, UK; corinne.finn@nhs.net (C.F.-F.); clare.dempsey@manchester.ac.uk (C.D.); 4 The Christie NHS Foundation Trust, Manchester M20 4BX, UK

**Keywords:** radiotherapy, polypharmacy, radiotherapy–drug interaction, metformin, diabetes mellitus, cancer survival outcome, adverse effects

## Abstract

**Background**: It remains unclear whether metformin, a widely used antidiabetic medication, has any influence on the survival outcomes or treatment-related toxicities of radiotherapy in cancer patients. Given metformin’s potential anti-cancer properties, including its ability to inhibit tumour growth through the modulation of cellular metabolism and enhancement of radiosensitivity, its impact on radiotherapy outcomes warrants thorough investigation. This study aimed to evaluate the impact of metformin on survival and adverse events among cancer patients receiving radiotherapy. **Methods**: Database searches were conducted in MEDLINE, EMBASE, Web of Science, Scopus, and PubMed (2000–2025) to retrieve studies of adults with cancer treated with radiotherapy and concurrent metformin. Metformin users were compared with non-users. The pooled overall survival rate was presented in terms of odds ratio (OR) and 95% confidence interval (95%CI). Diabetic subgroup analyses and meta-regression by cancer type were conducted. ORs and 95%CIs of radiotherapy-related adverse events were presented by cancer type. **Results**: This study identified 25 articles. The pooled overall survival rate showed no significant difference between metformin users and non-users across subgroups (ORs: 1.00–1.77). Conflicting survival trends were observed for prostate, oesophageal, and non-small cell lung cancer across diabetic conditions. Metformin users with breast cancer exhibited a significantly lower risk of heart failure (OR: 0.72; 95%CI: 0.56–0.94) and heart events (OR: 0.72; 95%CI: 0.59–0.88). **Conclusions**: Metformin did not significantly impact overall survival but may reduce heart-related adverse events in breast cancer patients based on limited data. Further research is needed on cancer types and diabetic conditions.

## 1. Introduction

Metformin is the first-line oral treatment for type 2 diabetes mellitus (T2DM) [1], the most common type of DM, accounting for 90–95% of DM cases [2]. Metformin reduces hepatic glucose production, enhances insulin sensitivity, and decreases DM-related morbidity and mortality [3,4,5,6,7]. Additionally, metformin may prevent cancer development [8,9,10] by activating 5′-adenosine monophosphate kinase, inhibiting the rapamycin pathway’s mammalian target, thereby reducing protein biosynthesis and tumour cell growth [11]. Thus, the idea of repurposing metformin, initially developed as an antidiabetic drug, has been proposed as an anti-cancer agent and evaluated through real-world evidence [12].

Radiotherapy is an essential cancer treatment [7], used in nearly 40% of patients with cancer [13]. However, thoracic radiotherapy for lung cancer can cause side effects, including pneumonitis [14] and oesophagitis [15,16] due to local inflammation. These side effects might be exacerbated by drugs such as metformin [17]. Given that metformin is a common medication taken by patients with T2DM [18] and has been studied in patients with cancer [12], its impacts on the tolerability and effectiveness of radiotherapy have been investigated in several observational cohort studies [11,19] and randomised controlled trials [20,21]. However, these studies reported controversial results. For example, a retrospective cohort study of 276 patients with glioblastoma and DM found that metformin users had significantly prolonged progression-free survival following radiotherapy [11]. Conversely, in another retrospective cohort study of 166 patients with locally advanced, inoperable non-small cell lung cancer (NSCLC) treated with definitive chemoradiotherapy, no significant difference in overall survival was found between metformin users and non-users (either diabetic or non-diabetic) [19].

In addition, the association between metformin use and radiotherapy-related adverse events varied by cancer type and stage. In a retrospective cohort study of 6993 patients with early-stage breast cancer receiving adjuvant breast radiotherapy, metformin users had a significantly reduced risk of radiation-induced cardiac toxicity [22]. However, in another retrospective cohort study of 252 patients with DM and stage III and IV head and neck squamous cell carcinoma receiving concurrent chemoradiotherapy, metformin users were significantly less likely to tolerate the chemotherapy agent. Although not statistically significant, they experienced more treatment-related toxicities compared to non-users [23].

The controversial evidence regarding the effects of metformin use in patients receiving radiotherapy may be attributed to variations in other treatments given in combination with radiotherapy, cancer prognoses, and study biases. Currently, there is no consensus on the impact of metformin on outcomes and adverse events in diabetic or non-diabetic patients during radiotherapy. It remains unclear in clinical oncology whether the concurrent use of metformin with radiotherapy raises any additional concerns regarding survival outcomes or the toxicities of radiotherapy. Therefore, this systematic review and meta-analysis aimed to investigate the existing evidence on the impact of metformin on survival outcomes and adverse events in patients with cancer undergoing radiotherapy, thereby clarifying its role in cancer treatment.

## 2. Materials and Methods

This systematic review and meta-analysis followed the Preferred Reporting Items for Systematic Reviews and Meta-Analyses (PRISMA) statement guidelines [24] (Supplemental S1). The protocol has been registered at PROSPERO (no. CRD42023487336).

### 2.1. Selection Criteria

This study’s inclusion and exclusion criteria are summarised as follows (Table 1).

### 2.2. Types of Studies

Prospective or retrospective cohort studies, cross-sectional studies, and clinical trials were included, whilst case–control studies, case series, case reports, systematic reviews, meta-analyses, conference abstracts, editorials, letters to editors, commentary, and grey literature were excluded.

### 2.3. Types of Participants

Studies enrolled participants aged eighteen and above with histologically confirmed cancer scheduled for radiotherapy. Exclusions were studies with participants under eighteen, mixed age groups, individuals undergoing neoadjuvant radiotherapy or diagnostic radiology, and cancers not indicated for radiotherapy.

### 2.4. Types of Interventions

Patients who received metformin orally, either as monotherapy or in combination with other medications (e.g., chemotherapy), during the course of radiotherapy were included. Patients not using metformin concurrently during radiotherapy were excluded.

### 2.5. Types of Outcome Measures

Studies evaluating survival outcomes and radiotherapy-related side effects were included. Survival outcomes encompassed survival or recurrence results. Radiotherapy-related adverse events occurring during or immediately after radiotherapy were included. Any radiation-related toxicity that occurred before the administration of metformin was not considered.

### 2.6. Data Sources and Search Strategies

A comprehensive electronic database search was conducted on MEDLINE, EMBASE, Web of Science, Scopus, and PubMed from January 2000 to 10 August 2025. The search start date was determined based on a preliminary search indicating that relevant literature emerged after 2000. Various structured search strategies were employed (Supplemental S2), utilising controlled vocabulary and keywords aligned with the study’s inclusion and exclusion criteria (Table 1). Additionally, the search was restricted to articles published in English and human studies.

### 2.7. Study Selection

Two reviewers (WCL and HS) independently screened titles and abstracts of articles retrieved from electronic database searches based on predefined selection criteria (Table 1) using a pre-designed electronic screening form. Articles were categorised as “included,” “further check,” or “excluded.” Consistency between reviewers was assessed using the intraclass correlation coefficient (two-way mixed effect model with absolute agreement) with a 95%CI [25]. Any discrepancies were resolved through discussion between reviewers and, if necessary, with a third reviewer (LCC) to achieve consensus. Potentially eligible articles underwent further independent review by both reviewers (WCL and HS) to determine study inclusion.

### 2.8. Data Extraction and Management

Two reviewers (WCL and HS) independently extracted data from each study using a standardised and piloted electronic data extraction sheet. Discrepancies were resolved by a third reviewer (LCC). Extracted information included the study title, lead author, country, publication year, study design, setting, targeted population (disease and cancer stages), intervention (metformin exposure), comparison, outcome measures, and follow-up period. Study results were retrieved, including the number of numerator and denominator of survival outcomes and the proportion of adverse events during or immediately after radiotherapy. If raw data were unavailable, the mean (with standard deviation) or median (range) of survival duration, or any other results convertible into raw data, were extracted.

### 2.9. Risk of Bias Assessment

Quality assessment of all included studies was conducted using the Cochrane Risk of Bias Assessment Tool (RoB 2) for randomised controlled trials (RCTs) and the Risk of Bias in Non-randomised Studies of Interventions tool (ROBINS-I) for non-randomised studies [26,27]. Studies were categorised into low risk of bias, some concerns, or high risk of bias according to RoB 2, as well as low, moderate, serious, or critical risk of bias based on ROBINS-I. The findings were summarised and tabulated.

### 2.10. Data Analysis

All outcomes were compared between metformin-exposed (users) and non-exposed (non-users) groups, with consideration of diabetic status categorised into five groups, including “not specified (NS)”, indicating mixed populations (Supplemental S1). Overall survival rates were prioritised, with years closest to five years censored if reported at multiple periods, considering the five-year survival rate is widely recognised as an essential metric of cancer care quality and long-term results [28].

The primary outcome is pooled effect sizes for overall survival rates presented as ORs with 95%CIs. Radiotherapy-related adverse events were tabulated with OR (95%CI) and categorised by cancer type in pooled results. Effect sizes for survival outcomes and radiotherapy-related adverse events were synthesised using a random-effects model (Der-Simonian and Laird method) [29]. Heterogeneity was assessed with the *I*^2^ test, supplemented by meta-regression to explore effects by cancer type.

Other survival rate outcomes were presented by OR and 95%CI across groups. Pooled effect sizes (OR and 95%CI) were calculated for progression-free, distant metastasis-free, disease-free survival, distant metastasis, locoregional recurrence, and biochemical failure rates when data were available.

Meta-analyses and meta-regression were conducted using STATA (Release 14, College Station, TX, USA: StataCorp LLC). A *p*-value < 0.05 was considered statistically significant.

## 3. Results

### 3.1. Selection of Study

**Figure 1:**
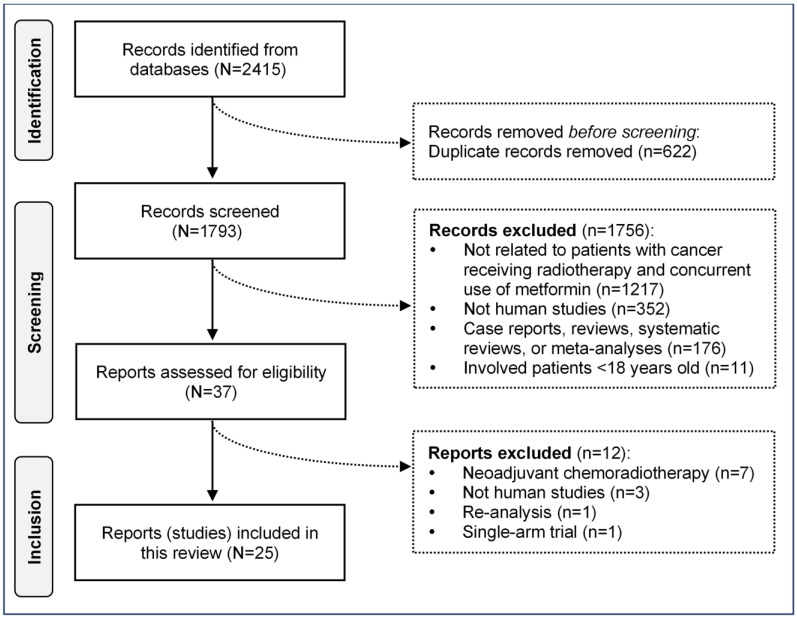
Selection of studies.

Of the 2415 records identified, 622 duplicates and 1756 irrelevant records were removed, leaving 37 articles for full-text screening. Exclusions were due to unrelated topics (n = 1217), non-human studies (n = 352), non-original studies (n = 176), and studies involving non-adult populations (n = 11). Twelve articles were excluded, resulting in 25 studies (20,953 patients) analysed (Figure 1). The intraclass correlation coefficient between the two reviewers was 0.983 (95% confidence interval [95%CI]: 0.982, 0.984), indicating good consistency.

### 3.2. Characteristics of Included Studies

The 25 included studies comprised 20 cohort studies [11,19,22,23,30,31,32,33,34,35,36,37,38,39,40,41,42,43,44,45] and five randomised trials [20,21,46,47,48] investigating various cancers: prostate cancer (n = 8) [30,31,34,35,36,38,41,46], NSCLC (n = 7) [19,20,21,40,45,47,48], breast cancer (n = 2) [22,32], and eight other cancers [11,23,33,37,39,42,43,44] (Table 2). Most studies specified metformin use in diabetic patients (n = 16) [11,19,23,31,32,33,35,36,37,38,39,40,41,43,44,45], followed by non-diabetic patients (n = 6) [20,21,32,46,47,48] and unspecified diabetic conditions, where the authors did not expressly state whether patients had DM (n = 4) [22,30,34,42] (Supplemental S3). Non-users of metformin were predominantly non-diabetic patients (n = 16) [11,19,20,21,31,33,37,38,39,40,41,43,44,46,47,48], followed by diabetic patients who could be on other antidiabetic medications (n = 11) [11,19,32,33,37,38,39,40,41,43,44] and those with unspecified diabetic conditions (n = 8) [22,23,30,34,35,36,42,45]. Fifteen research articles reported overall survival rates [19,21,23,30,31,33,37,38,39,42,43,44,45,47,48], whilst nine studies detailed radiotherapy-related side effects in patients with the following cancers: prostate cancer (n = 3) [30,31,46], NSCLC (n = 3) [20,21,47], breast cancer (n = 2) [22,32], and head and neck squamous cell carcinoma (SCC) (n = 1) [23].

### 3.3. Quality Assessment

Among the five RCTs, three were flagged for potential bias related to varying cancer stages influencing the outcome (n = 3) [20,46,48] and missing outcome data due to patient withdrawal and loss of follow-up (n = 3) [20,46,47]. Additionally, two RCTs were considered to have a high risk of bias due to deviations from the intended intervention to medication compliance issues (n = 2) [21,47], bias in the randomisation process (n = 1) [21], and selective reporting (n = 1) [21] (Supplemental S4).

Using the ROBINS-I tool, ten cohort studies had a serious risk of bias [11,23,32,33,34,36,38,40,41,42], and ten were classified with a moderate risk of bias. Those ten categorised with a serious risk of bias primarily lacked reporting on radiation dose (n = 5) [34,36,38,41,42], follow-up period (n = 5) [11,23,32,34,42], cancer stage (n = 3) [11,33,34], or age of the population (n = 2) [33,42]. In all cohort studies, confounding factors such as cancer stages, DM status, dose and duration of metformin, and potential systemic conditions were regarded as having a moderate risk of bias (Supplemental S5).

### 3.4. Overall Survival Rates

No significant differences were found in overall survival rates between metformin users and non-users across diabetic status categories (pooled odds ratio [OR] ranged from 1.00 to 1.77) despite heterogeneity in several groups (*I*^2^ ranged from 55.2% to 82.2%) (Table 3). When stratified by cancer type, the pooled OR was 1.26 (95%CI: 0.86, 1.83) for lung cancer and 1.67 (95%CI: 0.62, 4.49) for prostate cancer. Neither subgroup showed a statistically significant difference in overall survival rates between metformin users and non-users (Supplemental S6).

However, a significant correlation between overall survival and cancer type was observed in studies involving patients with unspecified diabetes mellitus (DM) status (Group 1, n = 5), with a meta-regression coefficient of β = −0.99 (95%CI: −1.95, −0.02; *p* < 0.05). In this group, metformin users diagnosed with hepatocellular carcinoma and oesophageal cancer showed improved survival, whereas those with prostate or cervical cancer had poorer outcomes. Similarly, in studies comparing metformin users with DM to non-users without DM (Group 4, n = 5), overall survival was significantly associated with cancer type (β = −0.60; 95%CI: −1.00, −0.19; *p* = 0.02). In this subgroup, metformin users with hepatocellular carcinoma and prostate cancer had better survival. In contrast, those with rectal cancer, oropharyngeal cancer, and non-small cell lung cancer (NSCLC) had worse outcomes.

No significant correlation with cancer type was found in studies where both metformin users and non-users had DM (Group 3, n = 6). For Group 2 (metformin users with DM vs. non-users with unspecified DM status), only two studies were available, which were insufficient for meta-regression analysis. Group 5 included only NSCLC studies comparing metformin users and non-users without DM (n = 3), limiting the ability to assess cancer-type correlations in this subgroup. 

### 3.5. Cancer Progression Outcomes

Likewise, substantial heterogeneity and no significant differences were found between metformin users and non-users in the pooled progression-free (*I*^2^ = 62.6%), distant metastasis-free (*I*^2^ = 88.2%), and disease-free survival rates; distant metastasis rate (*I^2^* = 70.2%); locoregional recurrence rate; and biochemical failure rate (*I*^2^ = 94.9%) (Table 4).

In one study of NSCLC, metformin users with DM had a significantly lower progression rate (OR: 0.50; 95%CI: 0.29, 0.87) compared to non-users with unspecified DM condition [45] (Supplemental S7). Median progression-free survival was longer in metformin users with DM than in non-users with glioblastoma [11] and non-users with DM [19] or unspecified condition [45] with NSCLC (range: 3.43, 26). Conversely, compared to non-users without DM [19,40] or unspecified DM [40] condition, metformin users with DM [19,40] or unspecified DM [40] had a shorter median progression-free survival time in NSCLC (range: −12.5, −1.9). Metformin users with DM presented a shorter median locoregional recurrence-free (range: −3.6, −2.2) [19] and distant metastasis-free survival time (range: −7.4, −3.4) [19] when compared to non-users with or without DM and NSCLC (Supplemental S7).

Significant correlations with cancer type were observed only in the distant metastasis-free survival rates (β: −1.68; 95%CI: −2.26, −1.10; *p* = 0.01), where metformin use presented significantly better outcomes in patients with NSCLC but worse outcomes in those with oropharyngeal cancer, oesophageal cancer, and prostate cancer, regardless of reported DM status (n = 4). Notably, cancer-type correlation analysis was impossible for the biochemical failure rate, as only studies in the field of prostate cancer reported this outcome.

### 3.6. Other Survival Outcome Measures

Metformin users with DM presented a significantly lower overall prostate cancer mortality rate than non-users with (OR: 0.22; 95%CI: 0.13, 0.37) or without DM (OR: 0.67; 95%CI: 0.46, 0.97) [41]. Other survival outcomes generally did not show significant differences, with some presenting wide 95%CIs (Supplemental S6). The mean prostate-specific antigen was lower in metformin users with DM (mean ± standard deviation: 5.4 ± 2.1) than in non-users with unspecified DM conditions (mean ± standard deviation: 9.2 ± 7.9) of prostate cancer [35].

Median survival times varied across studies. Metformin users with DM had a longer median survival time than non-users with unspecified conditions and NSCLC (n = 1) [45]. However, in another study, including patients with NSCLC, the median survival time was shorter in metformin users with DM than non-users with or without DM (range: −4.9, −2) [19] (Supplemental S7).

### 3.7. Radiotherapy-Related Adverse Events

The pooled results were presented if the same adverse events were reported in different studies. However, most of them were elucidated separately and diversely with OR and 95%CI by a single study. Overall, there was no significant difference between metformin users and non-users in the pooled results of acute gastrointestinal toxicity in patients with prostate cancer. In addition, there was no significant difference between metformin users and non-users in grade ≥3 nausea and vomiting in patients with head and neck SCC and NSCLC (Table 5). Nonetheless, metformin users with a diagnosis of breast cancer had a significantly lower risk of heart failure (OR: 0.72; 95%CI: 0.56, 0.94) and all heart events (OR: 0.72; 95%CI: 0.59, 0.88) when compared to non-users with unspecified DM condition. Metformin users with DM and a diagnosis of head and neck SCC experienced more significant weight loss compared to non-users with unspecified DM conditions [23].

## 4. Discussion

This study investigated whether concurrent metformin use positively or negatively impacts survival outcomes and adverse events in patients with cancer receiving radiotherapy, aiming to inform oncology practices. Based on the current clinical evidence, there was no significant difference in overall survival rates between metformin users and non-users, even when categorising them into different diabetes mellitus status subgroups. However, the type of cancer influenced survival rates differently across these subcategories, suggesting that the DM condition might affect them. Interestingly, within the limited adverse event reports, metformin showed significant benefits in reducing radiotherapy-related adverse events, particularly in heart failure and all heart events among patients with breast cancer.

The conflicting results regarding the effect of metformin on cancer radiotherapy outcomes in our study align with findings from past research [49,50]. The effect of metformin use on different cancer types has been addressed. The progression-free survival (PFS) rates were reported to be better for metformin users with reproductive cancers (breast, ovary, endometrium, and prostate) but worse for those with digestive cancers (pancreatic and liver) [51]. A meta-analysis published in 2016 investigating the association between metformin use and cancer outcomes in diabetic patients undergoing curative-intent cancer treatments suggested that metformin could provide clinical benefit, particularly in patients with colorectal and prostate cancer receiving radical radiotherapy [52]. In contrast to the previous meta-analysis, we aimed to clarify the DM condition in both metformin users and non-users through our data analysis. As we observed conflicting survival trends for the same type of cancer (prostate cancer, oesophageal cancer, and NSCLC) across different DM conditions between the metformin users and non-users in this study, these contrasting results underscore the need for further clarification regarding the interplay between cancer type and DM status of the patients.

The unexpected lack of a consistently significant effect of metformin in observational cohort studies and RCTs may have resulted from unavoidable confounding factors or variations among the studied participants. As substantial heterogeneity exists among the included studies, this may mask metformin’s efficacy due to differences in cancer types, grades, and stages [19,41,52]. Variations in DM condition, control status, and other underlying cardiovascular diseases could influence the impact of metformin on outcome [19,40]. Differences in metformin dose, duration of exposure, and inconsistent reporting in some studies could lead to conflicting estimates [19,49,53]. Furthermore, the optimal follow-up period for survival assessment differs among cancer types [54]. The use of overall survival rates as an outcome measure makes it difficult to determine the cause of death and whether patients die from cancer or other comorbidities. In addition, combined results may be influenced by the number of studies for each tumour type [52]. Adverse effects such as diarrhoea could affect medication adherence, posing challenges in retrospective study monitoring [51]. Moreover, metformin’s beneficial effect may apply only to specific tumourigenic mutations [55]. These factors may contribute to the observed discrepancies and explain why consistent results have not been reached in past studies.

Rates of adverse events did not significantly differ between metformin users and non-users in this study. Only patients with early-stage breast cancer showed a reduced risk of heart failure and all heart events in one retrospective cohort study [22]. In contrast, increased toxic effects and treatment breaks among metformin users have been reported to limit the dose and effectiveness of chemoradiotherapy [21,32]. This might be explained by the fact that metformin and concurrent chemoradiotherapy have been found to result in similar adverse effects, such as nausea, vomiting, diarrhoea, abdominal pain, and indigestion, which could lead to increased toxicity [23]. Considering that side effects are frequently reported by patients, it can sometimes be difficult for them to determine whether the symptoms originate from radiotherapy, metformin, or DM, and the adverse events may result from a combination of these factors [31]. As DM has been associated with increased tissue damage, reduced immune function, and endothelial dysfunction, which can exacerbate impairment after radiotherapy [31], clinicians should be aware of the potential for increased side effects in metformin users with DM during radiotherapy and provide additional supportive care to these patients [23].

This study’s strengths lie in its comprehensive search and inclusion of current evidence on metformin’s effect in patients undergoing radiotherapy for various cancers, allowing for pooled result analysis. We analysed 25 studies (20 retrospective cohort studies and five RCTs), surpassing a previous meta-analysis from 2018, which included only 17 retrospective cohort studies and DM patients [56]. To mitigate confounding factors of DM, we categorised metformin users and non-users into different groups for overall survival rate analysis, although significant findings were not observed. Despite the inability to demonstrate a clear benefit of metformin on survival outcomes and radiotherapy-related side effects, our findings suggest directions for further investigation. Based on current clinical evidence, this study contributes to understanding metformin’s potential interaction with radiotherapy, suggesting that the type of cancer may influence its effects.

The primary limitation of this study is the inclusion of mainly retrospective observational cohort studies, which are susceptible to bias in confounding and selection of reported results. This poses a moderate to serious risk of bias and may influence the outcomes. However, five RCTs were included; two were identified as having a high risk of bias, and small sample sizes with missing outcome data introduce uncertainty into the interpretation. Additionally, all participants in the metformin user and non-user groups of these trials did not have DM, which distinguished them from other observational cohort studies regarding patients’ characteristics. Although subgroup analyses were conducted by DM status and cancer type, stratification by study design (i.e., separating RCTs from cohort studies) was not feasible due to limited sample sizes. Future studies with larger datasets should consider this approach to better address heterogeneity and improve interpretability.

Most studies included in this meta-analysis identified patients as having DM as well as reported metformin use, but few provided details on the timing of DM diagnosis or the sequence of antidiabetic treatments. The lack of consistent reporting on these factors may contribute to heterogeneity in outcomes and limit the ability to fully understand metformin’s effects in the context of DM duration and treatment lines. In addition, cardiovascular comorbidities or specific treatments related to macrovascular complications among patients with DM could impact the results. Still, they could not be thoroughly investigated due to unavailable data, which were not consistently reported in the included literature. Since the included studies mainly focused on radiotherapy outcomes and did not elucidate patient characteristics regarding glycaemic control or other metabolic parameters, the specific effects of metformin, independent of overall DM management, could not be thoroughly assessed.

Despite our best efforts to consider patients’ DM status by grouping them, only one type of cancer (NSCLC) was included in group 5, where both metformin users and non-users were without DM. This limited the analysis of the prophylactic effect of metformin in non-diabetic patients compared to non-users across different types of cancer. Besides, more studies focused on two types of common cancers, prostate cancer (n = 8) and NSCLC (n = 7), potentially affecting the analysis results. Divergent reporting of adverse events also precluded meta-analysis for pooled results. Finally, this study is constrained by the data and indicators reported in the included studies.

The diverse and sometimes conflicting results from past research on metformin’s effect on patients with cancer undergoing radiotherapy highlight the need for more rigorous and well-structured future studies. RCTs offer maximum control over confounding factors but present challenges relating to cost, ethical considerations, and participant recruitment [57]. Therefore, further well-designed observational cohort studies are recommended, with predefined inclusion and exclusion criteria, to control for cardiovascular disease and DM status. Propensity score matching could be integrated into the methodology to mitigate challenges relating to confounding factors. Given the lack of a robust biomedical and pharmacological mechanism to explain the effects of metformin in combination with radiotherapy, the need to investigate the effects of metformin in specific phenotypic or genotypic settings has also been suggested [12].

As the impact of metformin in the context of cancer treatment remains uncertain, the challenges of polypharmacy in patients with cancer highlight the lack of evidence to guide oncology practice. Studying interactions between different treatments, particularly drugs and radiotherapy, is complex. This study will help guide discussions with patients. Based on the presented evidence, diabetic patients with cancer taking metformin and treated with radiotherapy are not expected to experience a significant impact on the outcome of the medication. For non-diabetic patients with cancer treated with radiotherapy, there is currently insufficient evidence to support metformin prophylaxis for improving survival outcomes or mitigating radiotherapy-related side effects.

## 5. Conclusions

In conclusion, this study found that metformin had no significant impact on radiotherapy survival outcomes regardless of patients’ DM status. Metformin may reduce heart-related adverse events in patients with breast cancer, but this finding is based on limited data and should be interpreted cautiously. Whilst the type of cancer may influence the overall survival rate between metformin users and non-users, inconsistent findings under different DM conditions warrant further research.

## Figures and Tables

**Table 1:** Inclusion and exclusion criteria of this study.

Component	Inclusion Criteria	Exclusion Criteria
Population and conditions	Patients aged 18 years and above. Patients diagnosed with histologically confirmed cancer (newly diagnosed or recurrent) are scheduled to receive radiotherapy.	Patients include paediatrics, children, adolescents, neonates, and infants. Studies involved mixed-age groups. Neoadjuvant radiotherapy or diagnostic radiology (e.g., X-rays, magnetic resonance images). Patients with cancer types not amenable to radiotherapy.
Intervention and comparator	Oral administration of metformin, either alone or in combination with other drugs, such as chemotherapy.	Non-concurrent use of metformin and radiotherapy (not during the radiotherapy cycles).
Outcome	Survival outcomes included overall survival and other related results. Adverse events occurred during or right after the radiotherapy.	Radiation-related toxicity occurred before the administration of metformin.
Study type	Human studies.	Animal or in vitro studies.
Language	English.	Other languages without English translation.
Publication	Full-text article on prospective or retrospective cohort study, cross-sectional study, and clinical trial.	Case-control study, case series, case report, systematic review, meta-analysis, conference abstract, abstract without full article, editorial, letter to editors, commentary, and grey literature.

**Table 2:** Characteristics of included studies.

Author, Year, Country	Cancer	Types of Radiation	Radiation Dose (Gy)	Number of Patients					Age of Patients (Year)	Outcome Category
				Total	Metformin Users		Non-User			
					DM (+)	DM (−)	DM (+) ^§^	DM (−)		
Ferro, 2013, US [32]	Breast cancer	WBI or external-beam partial-breast irradiation	Range: 45, 50	130	51	51	28		Mean (range): 60 (27, 83)	RT-related side effects
Skinner, 2013, US [37]	Rectal cancer	CRT	Median (range): 50.4 (20, 63)	482	20		40	422	Median (range): 58 (19, 84)	Survival outcomes
Spratt, 2013, US [38]	Prostate cancer	EBRT	NA	2901	157		162	2582	Median (IQR): 69 (64, 73)	Survival outcomes
Taira, 2014, US [41]	Prostate cancer	Interstitial brachytherapy	NA	2298	126		144	2028	Mean ± SD: 65.3 ± 7.4	Survival outcomes
Adeberg, 2015, Germany [11]	Primary glioblastoma	RT	Median (range): 60.0 (36.0, 68.0)	276	20		20	236	Median (range): 63.0 (17.2, 86.6)	Survival outcomes
Ahmed, 2015, US [19]	NSCLC	Thoracic RT	Median (range): 62 (60, 66)	166	20		20	126	Median (range): 65 (24, 86)	Survival outcomes
Jang, 2015, Korea [33]	Hepatocellular carcinoma	SBRT or hypofractionated RT	Range: 25, 60	217	19		29	169	NA	Survival outcomes
Van De Voorde, 2015, Netherlands [44]	Oesophageal cancer	EBRT	Median (NA): 41.4 (NA) or 50.4 (NA)	196	19		5	172	Mean (range): 63 (37, 82)	Survival outcomes
Spratt, 2016, US [39]	Oropharyngeal cancer	EBRT	Median (IQR): 69.96 (66, 70)	1745	102		82	1561	Median (range): 56 (25, 91)	Survival outcomes
Wink, 2016, Germany [45]	Locally advanced NSCLC	CRT	Median (range): 66.1 (50, 129.6)	682	59			Non-user ^¶^: 623	Median (range): 63 (29, 87)	Survival outcomes
Chang, 2017, Taiwan [23]	Head and neck SCC	CRT	Range: 70, 74	252	39			Non-user ^¶^: 213	Median (range): 55 (26, 83)	Survival outcomes RT-related side effects
Liu, 2017, US [35]	Prostate cancer	RT	Median (range): 2000–2005: 75.6 (NA); 2009–2012: 80.3 (NA)	381	27			Non-user ^¶^: 354	Mean ± SD: 74.4 ± 6.0	Survival outcomes
Takiuchi, 2017, US [42]	Cervical cancer	Whole pelvic RT	NA	478	Metformin user ^¦^: 41			Non-user ^¶^: 437	NA	Survival outcomes
Li, 2019, Taiwan [34]	Prostate cancer	RT	NA	567	Metformin user ^¶^: 180			Non-user ^¶^: 387	Mean ± SD: 71.8 ± 8.7	Survival outcomes
Ranasinghe, 2019, Australia [36]	Prostate cancer	EBRT	NA	2055	113			Non-user ^¶^: 1942	Median (range): 70 (54, 79)	Survival outcomes
Tsou, 2019, Taiwan [43]	Hypopharyngeal SCC	CRT	Range: 60, 70	141	49		43	49	Mean ± SD: 63.64 ± NA	Survival outcomes
Yu, 2019, Taiwan [22]	Early-stage breast cancer	Breast RT	Median (range): 50.4 (50, 59.4)	6993	Metformin user ^¦^: 2062			Non-user ^¶^: 4931	Median (range): metformin user: 59.89 (NA); non-user: 59.35 (NA)	RT-related side effects
Cadeddu, 2020, Spain [30]	High-risk prostate cancer	RT	Range: 72, 76	447	Metformin users ^¤^: 70			Non-user ^¤^: 377	Median (range): 70 (46, 83)	Survival outcomes RT-related side effects
Chun, 2020, US [20]	Squamous or adenocarcinoma NSCLC	SBRT	Median (range): peripheral: 50 (NA); central: 70 (NA)	15		14		1	Median (range): 73 (51, 84)	Survival outcomes RT-related side effects
Dağdelen, 2021, Turkey [31]	Prostate cancer	Radical RT	Median (range): 78 (70, 80)	94	22			72	Median (range): 69 (53, 88)	Survival outcomes RT-related side effects
Kim, 2021, Canada [46]	Prostate cancer	Prostate/pelvic RT	Range: 76, 78	81		39		42	Mean ± SD: 72 ± 7.1	RT-related side effects
Skinner, 2021, US [47]	Locally advanced NSCLC	3D conformal or intensity-modulated RT	Median (range): 60 (NA)	167		86		81	Median (range): 64 (43, 86)	Survival outcomes RT-related side effects
Stang, 2021, US [40]	Locally advanced NSCLC	SBRT	Range: 50, 60	120	31		11	78	Mean ± SD: 71.38 ± 9.09	Survival outcomes
Tsakiridis, 2021, Canada [21]	Locally advanced NSCLC	Chest RT	Range: 60, 63	54		26		28	Mean ± SD: 65.6 ± 7.6	Survival outcomes RT-related side effects
Tate, 2024, US [48]	NSCLC	SBRT	Median (range): peripheral: 50 (NA); central: 70 (NA)	15		14		1	Median (range): 73 (51, 84)	Survival outcomes

(Note) US: United States. NSCLC: non-small cell lung cancer. SCC: squamous cell carcinoma. WBI: whole breast irradiation. CRT: chemoradiotherapy. EBRT: external beam radiation therapy. RT: radiotherapy. SBRT: stereotactic body radiation therapy. 3D: 3-dimensional. NA: not available. IQR: Interquartile range. DM: diabetes mellitus. ^§^ Patients could be on other medications for diabetes mellitus. ^¶^ Includes patients with and without diabetes mellitus. ^¦^ May include patients with and without diabetes mellitus or all with diabetes mellitus. ^¤^ The article did not specify whether the patients had diabetes mellitus. SD: standard deviation.

**Table 3:** Overall survival rate outcome in five groups.

Study	Cancer	Comparison	Event Rate	Odds Ratio (95%CI)	Effect Size
**Group 1: metformin (NS) vs. non-user (NS)**					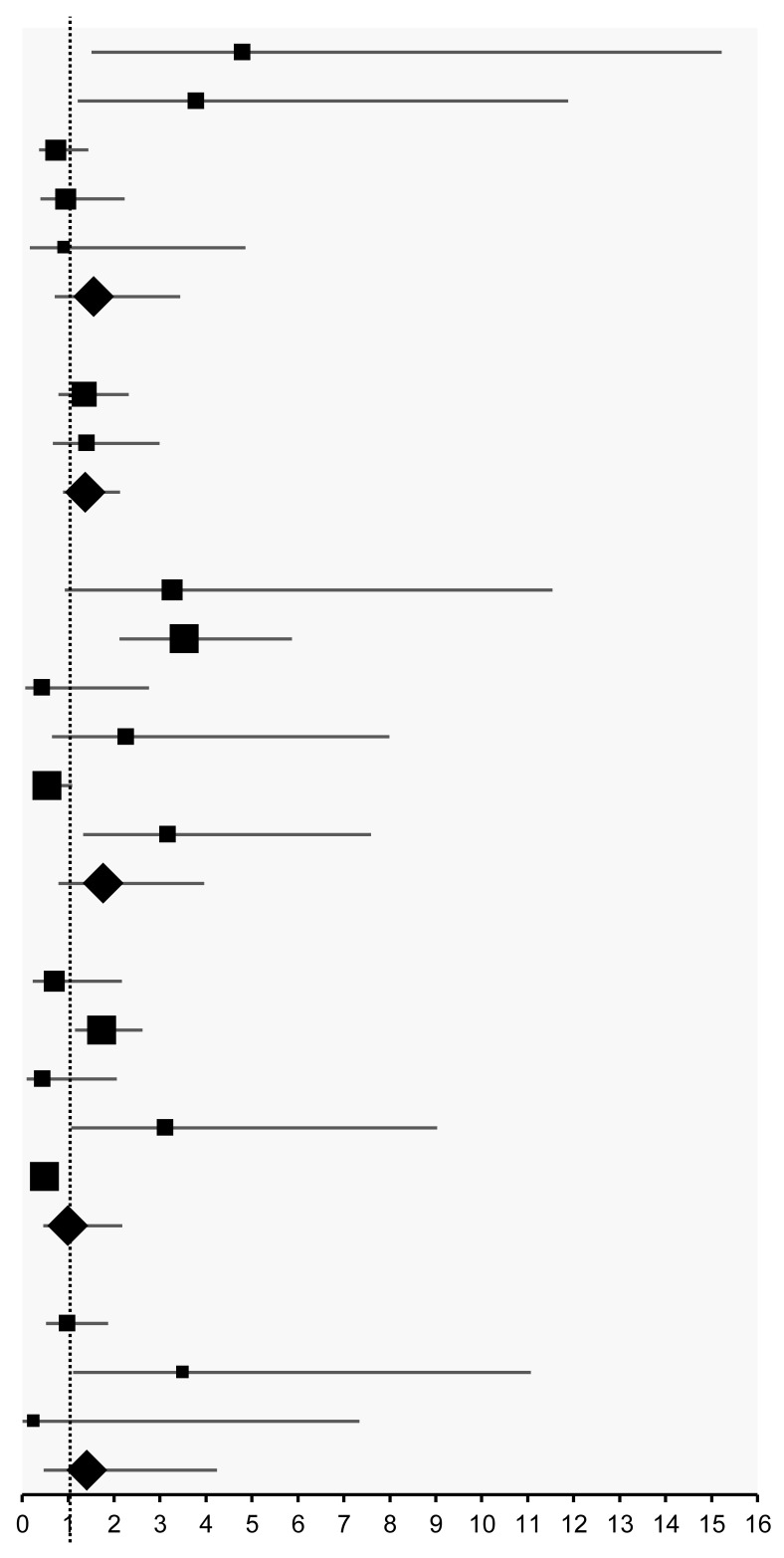
Jang (2015) [33]	Hepatocellular carcinoma	Metformin (NS) vs. non-user (NS)	14/19 vs. 21/57	4.80 (1.51, 15.22)	
Van De Voorde (2015) [44]	Oesophageal cancer	Metformin (NS) vs. non-user (NS)	15/19 vs. 88/177	3.79 (1.21, 11.88)	
Takiuchi (2017) [42]	Cervical cancer	Metformin (NS) vs. non-user (NS)	27/41 vs. 317/437	0.73 (0.37, 1.44)	
Cadeddu (2020) [30]	High-risk prostate cancer	Metformin (NS) vs. non-user (NS)	63/70 vs. 341/377	0.95 (0.40, 2.23)	
Dağdelen (2021) [31]	Prostate cancer	Metformin (NS) vs. non-user (NS)	20/22 vs. 66/72	0.91 (0.17, 4.86)	
**Overall**		**Metformin (NS) vs. non-user (NS)**	**139/171 vs. 833/1120**	**1.56 (0.71, 3.44), *I*^2^ = 66.0%**	
**Group2: metformin (DM+) vs. non-user (NS)**					
Wink (2016) [45]	Locally advanced NSCLC	Metformin (DM+) vs. non-user (NS)	33/59 vs. 301/623	1.36 (0.79, 2.32)	
Chang (2017) [23]	Head and neck SCC	Metformin (DM+) vs. non-user (NS)	28/39 vs. 137/213	1.41 (0.67, 2.99)	
**Overall**		**Metformin (DM+) vs. non-user (NS)**	**61/98 vs. 438/836**	**1.38 (0.89, 2.13), *I*^2^** ** < 0.1%**	
**Group 3: metformin (DM+) vs. non-user (DM+)**					
Skinner (2013) [37]	Rectal cancer	Metformin (DM+) vs. non-user (DM+)	16/20 vs. 22/40	3.27 (0.93, 11.54)	
Spratt (2013) [38]	Prostate cancer	Metformin (DM+) vs. non-user (DM+)	128/157 vs. 90/162	3.53 (2.12, 5.87)	
Ahmed (2015) [19]	NSCLC	Metformin (DM+) vs. non-user (DM+)	2/20 vs. 4/20	0.44 (0.07, 2.76)	
Jang (2015) [33]	Hepatocellular carcinoma	Metformin (DM+) vs. non-user (DM+)	14/19 vs. 16/29	2.27 (0.65, 7.99)	
Spratt (2016) [39]	Oropharyngeal cancer	Metformin (DM+) vs. non-user (DM+)	72/102 vs. 67/82	0.54 (0.27, 1.09)	
Tsou (2019) [43]	Hypopharyngeal SCC	Metformin (DM+) vs. non-user (DM+)	27/49 vs. 12/43	3.17 (1.33, 7.59)	
**Overall**		**Metformin (DM+) vs. non-user (DM+)**	**259/367 vs. 211/376**	**1.77 (0.79, 3.96), *I*^2^** ** = 77.8%**	
**Group 4: metformin (DM+) vs. non-user (DM−)**					
Skinner (2013) [37]	Rectal cancer	Metformin (DM+) vs. non-user (DM−)	16/20 vs. 359/422	0.70 (0.23, 2.17)	
Spratt (2013) [38]	Prostate cancer	Metformin (DM+) vs. non-user (DM−)	128/157 vs. 1854/2582	1.73 (1.15, 2.62)	
Ahmed (2015) [19]	NSCLC	Metformin (DM+) vs. non-user (DM−)	2/20 vs. 25/126	0.45 (0.10, 2.06)	
Jang (2015) [33]	Hepatocellular carcinoma	Metformin (DM+) vs. non-user (DM−)	14/19 vs. 80/169	3.12 (1.07, 9.03)	
Spratt (2016) [39]	Oropharyngeal cancer	Metformin (DM+) vs. non-user (DM−)	72/102 vs. 1296/1561	0.49 (0.31, 0.77)	
**Overall**		**Metformin (DM+) vs. non-user (DM−)**	**232/318 vs. 3614/4860**	**1.00 (0.46, 2.18), *I^2^*** ** = 82.2%**	
**Group 5: metformin (DM−) vs. non-user (DM−)**					
Skinner (2021) [47]	Locally advanced NSCLC	Metformin (DM−) vs. non-user (DM−)	56/86 vs. 53/81	0.99 (0.52, 1.87)	
Tsakiridis (2021) [21]	Locally advanced NSCLC	Metformin (DM−) vs. non-user (DM−)	14/26 vs. 7/28	3.50 (1.11, 11.07)	
Tate (2024) [48]	NSCLC	Metformin (DM−) vs. non-user (DM−)	6/14 vs. 1/1	0.25 (0.01, 7.34)	
**Overall**		**Metformin (DM−) vs. non-user (DM−)**	**76/126 vs. 61/110**	**1.41 (0.47, 4.24), *I^2^*** ** = 55.2%**	
				

(Note) NS: The condition of diabetes mellitus was not specified. DM: diabetes mellitus. NSCLC: non-small cell lung cancer. SCC: squamous cell carcinoma.

**Table 4:** Cancer progression outcomes.

Study	Cancer	Comparison	Event Rate	Odds Ratio (95%CI)	Effect Size
**Pooled progression-free**					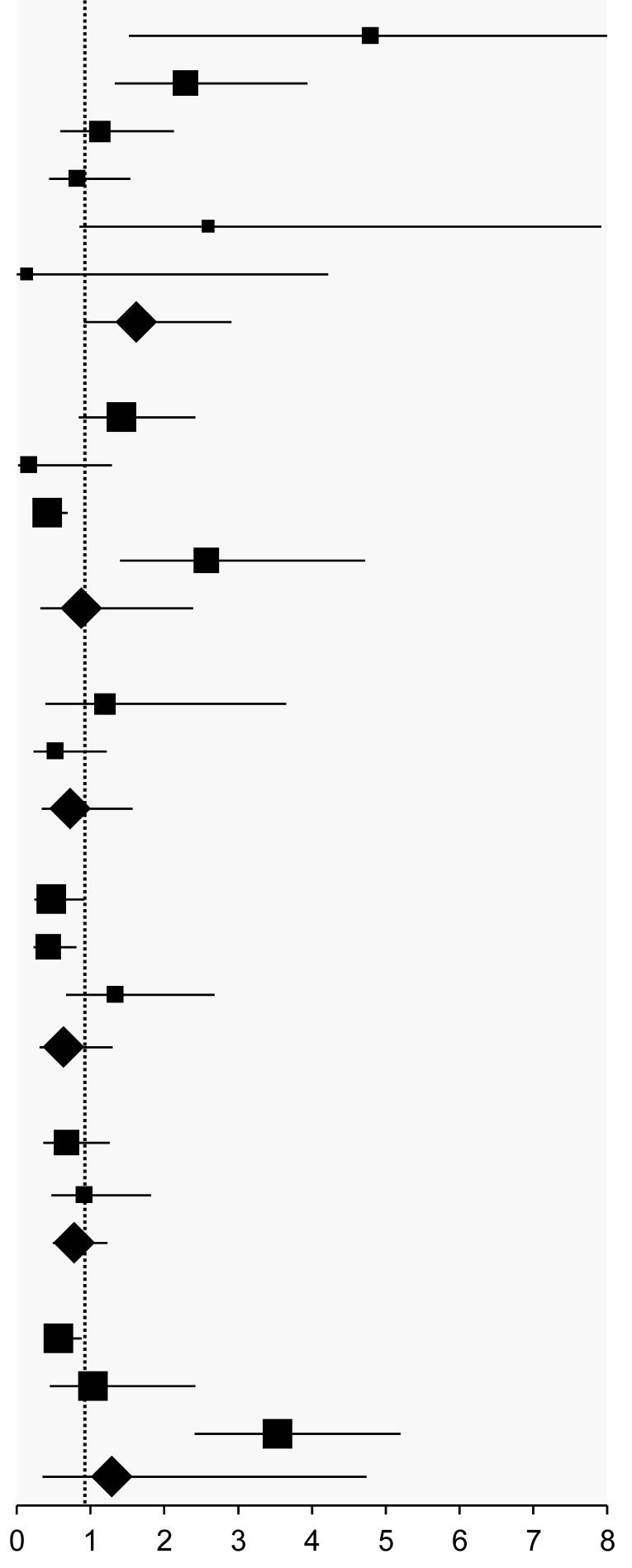
Jang (2015) [33]	Hepatocellular carcinoma	Metformin (NS) vs. non-user (NS)	9/19 vs. 9/57	4.80 (1.52, 15.13)	
Wink (2016) [45]	Locally advanced NSCLC	Metformin (DM+) vs. non-user (NS)	34/59 vs. 232/623	2.29 (1.33, 3.94)	
Takiuchi (2017) [42]	Cervical cancer	Metformin (NS) vs. non-user (NS)	23/41 vs. 233/437	1.12 (0.59, 2.13)	
Skinner (2021) [47]	Locally advanced NSCLC	Metformin (DM−) vs. non-user (DM−)	30/86 vs. 32/81	0.82 (0.44, 1.54)	
Tsakiridis (2021) [21]	Locally advanced NSCLC	Metformin (DM−) vs. non-user (DM−)	18/26 vs. 13/28	2.60 (0.85, 7.92)	
Tate (2024) [48]	NSCLC	Metformin (DM−) vs. non-user (DM−)	4/14 vs. 1/1	0.14 (0.00, 4.22)	
**Overall**			**118/245 vs. 520/1227**	**1.62 (0.91, 2.91), *I*^2^ = 62.6%**	
**Distant metastasis-free survival rate**					
Spratt (2013) [38]	Prostate cancer	Metformin (DM+) vs. non-user (DM−)	141/157 vs. 2223/2582	1.42 (0.84, 2.42)	
Van De Voorde (2015) [44]	Oesophageal cancer	Metformin (NS) vs. non-user (NS)	1/19 vs. 44/177	0.17 (0.02, 1.29)	
Spratt (2016) [39]	Oropharyngeal cancer	Metformin (DM+) vs. non-user (DM−)	80/102 vs. 1399/1561	0.42 (0.26, 0.69)	
Wink (2016) [45]	Locally advanced NSCLC	Metformin (DM+) vs. non-user (NS)	44/59 vs. 332/623	2.57 (1.40, 4.72)	
**Overall**			**266/337 vs. 3998/4943**	**0.88 (0.32, 2.39), *I*^2^ = 88.2%**	
**Disease-free survival rate**					
Skinner (2013) [37]	Rectal cancer	Metformin (DM+) vs. non-user (DM−)	16/20 vs. 325/422	1.19 (0.39, 3.65)	
Tsou (2019) [43]	Hypopharyngeal SCC	Metformin (DM+) vs. non-user (DM+)	22/49 vs. 26/43	0.53 (0.23, 1.22)	
**Overall**			**38/69 vs. 351/465**	**0.73 (0.34, 1.57), *I*^2^ = 22.3%**	
**Distant metastasis rate**					
Spratt (2013) [38]	Prostate cancer	Metformin (DM+) vs. non-user (DM−)	9/157 vs. 298/2582	0.47 (0.24, 0.92)	
Wink (2016) [45]	Locally advanced NSCLC	Metformin (DM+) vs. non-user (NS)	13/59 vs. 248/623	0.43 (0.23, 0.81)	
Skinner (2021) [47]	Locally advanced NSCLC	Metformin (DM−) vs. non-user (DM−)	25/86 vs. 19/81	1.34 (0.67, 2.68)	
**Overall**			**47/302 vs. 565/3286**	**0.64 (0.31, 1.30), *I*^2^ = 70.2%**	
**Locoregional recurrence rate**					
Wink (2016) [45]	Locally advanced NSCLC	Metformin (DM+) vs. non-user (NS)	14/59 vs. 196/623	0.68 (0.36, 1.26)	
Skinner (2021) [47]	Locally advanced NSCLC	Metformin (DM−) vs. non-user (DM−)	23/86 vs. 23/81	0.92 (0.47, 1.82)	
**Overall**			**37/145 vs. 219/704**	**0.78 (0.49, 1.23), *I*^2^ < 0.1%**	
**Biochemical failure rate**					
Spratt (2013) [38]	Prostate cancer	Metformin (DM+) vs. non-user (DM−)	26/157 vs. 666/2582	0.57 (0.37, 0.88)	
Taira (2014) [41]	Prostate cancer	Metformin (DM+) vs. non-user (DM−)	6/126 vs. 93/2028	1.04 (0.45, 2.42)	
Ranasinghe (2019) [36]	Prostate cancer	Metformin (DM+) vs. non-user (NS)	61/113 vs. 483/1942	3.54 (2.41, 5.20)	
**Overall**			**93/396 vs. 1242/6552**	**1.29 (0.35, 4.74), *I*^2^ = 94.9%**	
				

(Note) NSCLC: non-small cell lung cancer. SCC: squamous cell carcinoma. NS: The condition of diabetes mellitus was not specified. DM: diabetes mellitus.

**Table 5:** Radiotherapy-related adverse events.

Adverse Event	Study	Comparison	Follow-Up (Months)	Event Rate	Odds Ratio (95%CI)
**Prostate cancer**					
Acute gastrointestinal toxicity	Cadeddu (2020) [30]	Metformin (NS) vs. non-user (NS)	Median (range): 88 (1–194)	NA/70 vs. NA/377 ^¦^	1.00 (0.50, 1.60) ^†^
Acute gastrointestinal toxicity	Dağdelen (2021) [31]	Metformin (NS) vs. non-user (NS)	Median (range): 57 (15–128)	6/22 vs. 18/72	1.13 (0.38, 3.31) ^†^
Acute genitourinary toxicity	Cadeddu (2020) [30]	Metformin (NS) vs. non-user (NS)	Median (range): 88 (1–194)	NA/70 vs. NA/377 ^¦^	1.4 (0.30, 0.90) ^§^
Acute genitourinary toxicity	Dağdelen (2021) [31]	Metformin (NS) vs. non-user (NS)	Median (range): 57 (15–128)	20/22 vs. 54/72	3.33 (0.71, 15.68) ^§^
Chronic gastrointestinal toxicity	Cadeddu (2020) [30]	Metformin (NS) vs. non-user (NS)	Median (range): 88 (1–194)	NA/70 vs. NA/377 ^¦^	1.10 (0.30, 2.30)
Chronic genitourinary toxicity	Cadeddu (2020) [30]	Metformin (NS) vs. non-user (NS)	Median (range): 88 (1–194)	NA/70 vs. NA/377 ^¦^	1.0 (0.50, 1.80)
Late genitourinary side effects	Dağdelen (2021) [31]	Metformin (NS) vs. non-user (NS)	Median (range): 57 (15–128)	4/22 vs. 9/72	1.56 (0.43, 5.65)
Late gastrointestinal side effects	Dağdelen (2021) [31]	Metformin (NS) vs. non-user (NS)	Median (range): 57 (15–128)	3/22 vs. 3/72	3.63 (0.68, 19.47)
Overall genitourinary toxicity	Kim (2021) [46]	Metformin (DM−) vs. non-user (DM−)	Mean (range): 27.3 (0.5–63.2)	2/29 vs. 1/28	2.00 (0.17, 23.39)
Overall gastrointestinal toxicity	Kim (2021) [46]	Metformin (DM−) vs. non-user (DM−)	Mean (range): 27.3 (0.5–63.2)	1/29 vs. 0/28	2.00 (0.06, 62.06)
**Head and neck SCC**					
Feeding tube placement	Chang (2017) [23]	Metformin (DM+) vs. non-user (NS)	NA	29/39 vs. 125/213	2.04 (0.95, 4.40)
Toxic death (grade 5 toxicity)	Chang (2017) [23]	Metformin (DM+) vs. non-user (NS)	NA	2/39 vs. 9/213	1.23 (0.25, 5.90)
≥grade 3 mucositis/pharyngitis	Chang (2017) [23]	Metformin (DM+) vs. non-user (NS)	NA	16/39 vs. 116/213	0.58 (0.29, 1.16)
≥grade 3 nausea/vomiting	Chang (2017) [23]	Metformin (DM+) vs. non-user (NS)	NA	11/39 vs. 33/213	2.14 (0.97, 4.72) ^¶¤^
**Locally advanced NSCLC**					
≥grade 3 toxicities	Chun (2020) [20]	Metformin (DM−) vs. non-user (DM−)	Median (range): 6 (NA)	0/14 vs. 0/1	0.07 (0.00, 5.90)
≥grade 3 nausea	Skinner (2021) [47]	Metformin (DM−) vs. non-user (DM−)	Median (range): 27.7 (0.03–47.21)	2/86 vs. 1/81	1.90 (0.17, 21.42) ^¶^
≥grade 3 nausea	Tsakiridis (2021) [21]	Metformin (DM−) vs. non-user (DM−)	NA	0/26 vs. 1/28	0.52 (0.02, 16.15) ^¶^
≥grade 3 vomiting	Skinner (2021) [47]	Metformin (DM−) vs. non-user (DM−)	Median (range): 27.7 (0.03–47.21)	1/86 vs. 1/81	0.94 (0.06, 15.30) ^¤^
≥grade 3 vomiting	Tsakiridis (2021) [21]	Metformin (DM−) vs. non-user (DM−)	NA	0/26 vs. 1/28	0.52 (0.02, 16.15) ^¤^
≥grade 3 diarrhoea	Skinner (2021) [47]	Metformin (DM−) vs. non-user (DM−)	Median (range): 27.7 (0.03–47.21)	1/86 vs. 2/81	0.46 (0.04, 5.23)
≥grade 3 pneumonitis	Skinner (2021) [47]	Metformin (DM−) vs. non-user (DM−)	Median (range): 27.7 (0.03–47.21)	1/86 vs. 2/81	0.46 (0.04, 5.23)
≥grade 3 anaemia	Tsakiridis (2021) [21]	Metformin (DM−) vs. non-user (DM−)	NA	1/26 vs. 1/28	1.08 (0.06, 18.21)
≥grade 3 body odour	Tsakiridis (2021) [21]	Metformin (DM−) vs. non-user (DM−)	NA	1/26 vs. 0/28	2.24 (0.07, 69.68)
≥grade 3 dysphagia	Tsakiridis (2021) [21]	Metformin (DM−) vs. non-user (DM−)	NA	2/26 vs. 0/28	4.67 (0.20, 108.54)
≥grade 3 esophagitis	Tsakiridis (2021) [21]	Metformin (DM−) vs. non-user (DM−)	NA	0/26 vs. 1/28	0.52 (0.02, 16.15)
≥grade 3 lymphocyte count decreased	Tsakiridis (2021) [21]	Metformin (DM−) vs. non-user (DM−)	NA	5/26 vs. 1/28	6.43 (0.70, 59.28)
≥grade 3 neutrophil count decreased	Tsakiridis (2021) [21]	Metformin (DM−) vs. non-user (DM−)	NA	2/26 vs. 1/28	2.25 (0.19, 26.41)
≥grade 3 white blood cell count decreased	Tsakiridis (2021) [21]	Metformin (DM−) vs. non-user (DM−)	NA	3/26 vs. 1/28	3.52 (0.34, 36.22)
≥grade 3 respiratory failure	Tsakiridis (2021) [21]	Metformin (DM−) vs. non-user (DM−)	NA	1/26 vs. 0/28	2.24 (0.07, 69.68)
**Breast cancer**					
Treatment breaks secondary to skin toxicity	Ferro (2013) [32]	Metformin (DM+) vs. non-user (DM+)	NA	9/51 vs. 1/28	5.79 (0.69, 48.29)
Desquamation	Ferro (2013) [32]	Metformin (DM+) vs. non-user (DM+)	NA	28/51 vs. 9/28	2.57 (0.98, 6.75)
Heart failure	Yu (2019) [22]	Metformin (NS) vs. non-user (NS)	Mean ± SD: 61.68 ± 17.28	74/2062 vs. 241/4931	0.72 (0.56, 0.94) *
All heart events	Yu (2019) [22]	Metformin (NS) vs. non-user (NS)	Mean ± SD: 61.68 ± 17.28	129/2062 vs. 419/4931	0.72 (0.59, 0.88) *

(Note) SCC: squamous cell carcinoma. NSCLC: non-small cell lung cancer. NS: The condition of diabetes mellitus was not specified. DM: diabetes mellitus. NA: not available. SD: standard deviation. ^¦^ The case number was not provided, but the odds ratio and 95% confidence interval were reported in the included study. ^†^ Effect size: odds ratio: 1.03; 95% confidence interval: 0.62, 1.72; *I*^2^ < 0.1%. ^§^ Effect size: effect size and confidence interval limit are invalid. ^¶^ Effect size: odds ratio: 1.93; 95% confidence interval: 0.93, 4.02; *I*^2^ < 0.1%. ^¤^ Effect size: odds ratio: 1.84; 95% confidence interval: 0.88, 3.86; *I*^2^ < 0.1%. * Significantly lower risk was observed in metformin users compared to non-users when both groups were under unspecified diabetes mellitus conditions.

## Data Availability

No new data were created or analysed in this study.

## Supplementary Materials

The following supporting information can be downloaded at: https://www.mdpi.com/article/10.3390/ph18091390/s1, Supplemental S1: PRISMA 2020 Checklist; Supplemental S2: Electronic search strategy; Supplemental S3: Five comparison groups and the diabetic status of the patients in this study; Supplemental S4: The risk-of-bias assessment for randomised controlled trials; Supplemental S5: The risk-of-bias assessment for the cohort studies; Supplemental S6: Other survival rate outcomes; Supplemental S7: Survival time outcomes. Ref. [58] cited in Supplementary Materials.
