# Supplementary material for: Survival and Radiotherapy-Related Adverse Events in Patients Receiving Radiotherapy and Concurrent Metformin: A Systematic Review and Meta-Analysis of Randomised Controlled Trials and Cohort Studies
Source: Pharmaceuticals (Basel). 2025 Sep 17;18(9):1390. doi: 10.3390/ph18091390 (PMC12472684; doi:10.3390/ph18091390)
Supplement: Supplementary file 1 [file pharmaceuticals-18-01390-s001.zip › pharmaceuticals-3739096-supplementary.pdf]

Supplementary Material

Supplemental S1. PRISMA 2020 Checklist

Section and Topic	Item #	Checklist item	Location where item is reported
**TITLE**			
Title	1	Identify the report as a systematic review.	1
**ABSTRACT**			
Abstract	2	See the PRISMA 2020 for Abstracts checklist.	1-2
**INTRODUCTION**			
Rationale	3	Describe the rationale for the review in the context of existing knowledge.	2
Objectives	4	Provide an explicit statement of the objective(s) or question(s) the review addresses.	2
**METHODS**			
Eligibility criteria	5	Specify the inclusion and exclusion criteria for the review and how studies were grouped for the syntheses.	16-18, Table 5 and Appendix 6
Information sources	6	Specify all databases, registers, websites, organisations, reference lists and other sources searched or consulted to identify studies. Specify the date when each source was last searched or consulted.	18
Search strategy	7	Present the full search strategies for all databases, registers and websites, including any filters and limits used.	18, Appendix 6
Selection process	8	Specify the methods used to decide whether a study met the inclusion criteria of the review, including how many reviewers screened each record and each report retrieved, whether they worked independently, and if applicable, details of automation tools used in the process.	18-19
Data collection process	9	Specify the methods used to collect data from reports, including how many reviewers collected data from each report, whether they worked independently, any processes for obtaining or confirming data from study investigators, and if applicable, details of automation tools used in the process.	18-19
Data items	10a	List and define all outcomes for which data were sought. Specify whether all results that were compatible with each outcome domain in each study were sought (e.g. for all measures, time points, analyses), and if not, the methods used to decide which results to collect.	18-19
	10b	List and define all other variables for which data were sought (e.g. participant and intervention characteristics, funding sources). Describe any assumptions made about any missing or unclear information.	18-19
Study risk of bias assessment	11	Specify the methods used to assess risk of bias in the included studies, including details of the tool(s) used, how many reviewers assessed each study and whether they worked independently, and if applicable, details of automation tools used in the process.	18-19
Effect measures	12	Specify for each outcome the effect measure(s) (e.g. risk ratio, mean difference) used in the synthesis or presentation of results.	18-19
Synthesis methods	13a	Describe the processes used to decide which studies were eligible for each synthesis (e.g. tabulating the study intervention characteristics and comparing against the planned groups for each synthesis (item #5)).	18-19
	13b	Describe any methods required to prepare the data for presentation or synthesis, such as handling of missing summary statistics, or data conversions.	18-19
	13c	Describe any methods used to tabulate or visually display results of individual studies and syntheses.	18-19
	13d	Describe any methods used to synthesise results and provide a rationale for the choice(s). If meta-analysis was performed, describe the model(s), method(s) to identify the presence and extent of statistical heterogeneity, and software package(s) used.	18-19

Section and Topic	Item #	Checklist item	Location where item is reported
	13e	Describe any methods used to explore possible causes of heterogeneity among study results (e.g. subgroup analysis, meta-regression).	18-19, Appendix 7
	13f	Describe any sensitivity analyses conducted to assess robustness of the synthesised results.	18-19
Reporting bias assessment	14	Describe any methods used to assess risk of bias due to missing results in a synthesis (arising from reporting biases).	18-19
Certainty assessment	15	Describe any methods used to assess certainty (or confidence) in the body of evidence for an outcome.	18-19
**RESULTS**			
Study selection	16a	Describe the results of the search and selection process, from the number of records identified in the search to the number of studies included in the review, ideally using a flow diagram.	3, Figure 1
	16b	Cite studies that might appear to meet the inclusion criteria, but which were excluded, and explain why they were excluded.	3, Figure 1
Study characteristics	17	Cite each included study and present its characteristics.	3-4, Table 1
Risk of bias in studies	18	Present assessments of risk of bias for each included study.	8, Appendix 1 and 2
Results of individual studies	19	For all outcomes, present, for each study: (a) summary statistics for each group (where appropriate) and (b) an effect estimate and its precision (e.g. confidence/credible interval), ideally using structured tables or plots.	8-14, Table 2-4, Appendix 3 and 4
Results of syntheses	20a	For each synthesis, briefly summarise the characteristics and risk of bias among contributing studies.	8-14
	20b	Present results of all statistical syntheses conducted. If meta-analysis was done, present for each the summary estimate and its precision (e.g. confidence/credible interval) and measures of statistical heterogeneity. If comparing groups, describe the direction of the effect.	8-14, Table 2-4, Appendix 3 and 4
	20c	Present results of all investigations of possible causes of heterogeneity among study results.	8-14
	20d	Present results of all sensitivity analyses conducted to assess the robustness of the synthesised results.	8-14
Reporting biases	21	Present assessments of risk of bias due to missing results (arising from reporting biases) for each synthesis assessed.	8-14
Certainty of evidence	22	Present assessments of certainty (or confidence) in the body of evidence for each outcome assessed.	8-14
**DISCUSSION**			
Discussion	23a	Provide a general interpretation of the results in the context of other evidence.	14
	23b	Discuss any limitations of the evidence included in the review.	14-16
	23c	Discuss any limitations of the review processes used.	14-16
	23d	Discuss implications of the results for practice, policy, and future research.	14-16
**OTHER INFORMATION**			
Registration and	24a	Provide registration information for the review, including register name and registration number, or state that the review was not registered.	16

Section and Topic	Item #	Checklist item	Location where item is reported
protocol	24b	Indicate where the review protocol can be accessed, or state that a protocol was not prepared.	16-18
	24c	Describe and explain any amendments to information provided at registration or in the protocol.	16-18
Support	25	Describe sources of financial or non-financial support for the review, and the role of the funders or sponsors in the review.	19
Competing interests	26	Declare any competing interests of review authors.	19
Availability of data, code and other materials	27	Report which of the following are publicly available and where they can be found: template data collection forms; data extracted from included studies; data used for all analyses; analytic code; any other materials used in the review.	Not applicable

From: Page MJ, McKenzie JE, Bossuyt PM, Boutron I, Hoffmann TC, Mulrow CD, et al. The PRISMA 2020 statement: an updated guideline for reporting systematic reviews. BMJ 2021;372:n71. doi: 10.1136/bmj.n71

Supplemental S2. Electronic search strategy

MEDLINE

1 exp Radiotherapy/
2 exp Radiation/
3 exp Chemoradiotherapy/
4 exp Radiotherapy, Computer-Assisted/
5 Stereotactic body radiation therapy.mp.
6 Stereotactic radiosurgery.mp. or Radiosurgery/
7 Definitive Radiation Therapy.mp.
8 Thoracic Irradiation.mp.
9 Total body irradiation.mp. or Whole-Body Irradiation/
10 Radiotherap*.mp.
11 Radiother*.mp.
12 Radiat*.mp.
13 Irradiat*.mp.
14 (Radiochemo* or Chemoradio*).mp. [mp=title, book title, abstract, original title, name of substance word, subject heading word, floating sub-heading word, keyword heading word, organism supplementary concept word, protocol supplementary concept word, rare disease supplementary concept word, unique identifier, synonyms, population supplementary concept word, anatomy supplementary concept word]
15 Radiation therap*.mp.
16 1 or 2 or 3 or 4 or 5 or 6 or 7 or 8 or 9 or 10 or 11 or 12 or 13 or 14 or 15
17 exp Metformin/
18 Metformin*.tw.
19 (dimethylbiguanidium or dimethylguanylguanidine or glucophage or glucovance).tw.
20 17 or 18 or 19
21 16 and 20
22 limit 21 to (english language and humans and yr="2000 -Current" and "all adult (19 plus years)")

EMBASE

1 exp Radiotherapy/
2 exp Radiation/
3 exp Chemoradiotherapy/
4 exp Radiotherapy, Computer-Assisted/
5 Stereotactic body radiation therapy.mp.
6 Stereotactic radiosurgery.mp. or Radiosurgery/
7 Definitive Radiation Therapy.mp.
8 Thoracic Irradiation.mp.
9 Total body irradiation.mp. or Whole-Body Irradiation/
10 Radiotherap*.mp.
11 Radiother*.mp.
12 Radiat*.mp.
13 Irradiat*.mp.
14 (Radiochemo* or Chemoradio*).mp. [mp=title, abstract, heading word, drug trade name, original title, device manufacturer, drug manufacturer, device trade name, keyword heading word, floating subheading word, candidate term word]
15 Radiation therap*.mp.
16 1 or 2 or 3 or 4 or 5 or 6 or 7 or 8 or 9 or 10 or 11 or 12 or 13 or 14 or 15
17 exp Metformin/

18 Metformin*.tw.

19 (dimethylbiguanidium or dimethylguanylguanidine or glucophage or glucovance).tw.

20 17 or 18 or 19

21 16 and 20

22 limit 21 to (human and english language and yr="2000 -Current" and adult <18 to 64 years>)

Web of Science

(Radiotherapy or Radiation or Chemoradiotherapy or Stereotactic body radiation therapy or Stereotactic radiosurgery or Radiosurgery or Definitive Radiation Therapy or Thoracic Irradiation or Total Body Irradiation or Whole-Body Irradiation or Radiotherapy-Computer-Assisted or Radiotherap* or Radiother* or Radiat* or Irradiat* or Radiochemo* or Chemoradio* or Radiation therap*) and (Metformin or Metformin* or dimethylbiguanidium or dimethylguanylguanidine or glucophage or glucovance)

Scopus

(radiotherapy OR radiation OR chemoradiotherapy OR stereotactic OR stereotactic OR radiosurgery OR radiosurgery OR definitive OR thoracic OR total OR whole-body AND irradiation OR radiotherapy-computer-assisted OR radiotherap* OR radiother* OR radiat* OR irradiat* OR radiochemo* OR chemoradio* OR radiation AND therap*) AND (metformin OR metformin* OR glucophage OR glucovance)

PubMed

((radiotherapy) OR (radiation)) OR (chemoradiotherapy)) OR (radiosurgery)) OR (irradiation)) OR (radiotherapy-computer-assisted)) AND (metformin)

Supplemental S3. Five comparison groups and the diabetic status of the patients in this study

Group	Diabetic status		Label	Analytical outcomes
	Metformin users	Non-users		
Group 1	Not specified	Not specified	Metformin (NS) vs non-user (NS)	Overall survival rate<br>2-year local control rate<br>2-year intrahepatic metastasis-free survival rate<br>2-year extrahepatic metastasis-free survival rate<br>2-year survival rate after recurrence/progression<br>Biochemical failure-free survival rate<br>Disease-specific survival rate<br>Distant failure-free survival rate<br>Biochemical recurrence-free survival rate
Group 2	With DM	Not specified	Metformin (DM+) vs non-user (NS)	Overall survival rate<br>Distant relapse rate<br>Multifocal relapse rate<br>2-year locoregional recurrence-free survival rate<br>Progression rate<br>2-year recurrence-free survival rate
Group 3	With DM	With DM	Metformin (DM+) vs non-user (DM+)	Overall survival rate<br>Cause-specific mortality rate<br>Overall mortality rate<br>5-year local failure-free survival rate<br>5-year regional failure-free survival rate<br>Progression or mortality rate
Group 4	With DM	Without DM	Metformin (DM+) vs non-user (DM-)	Overall survival rate<br>Cause-specific mortality rate<br>Overall mortality rate<br>5-year local failure-free survival rate<br>5-year regional failure-free survival rate<br>Progression or mortality rate
Group 5	Without DM	Without DM	Metformin (DM-) vs non-user (DM-)	Overall survival rate<br>6-month progressive metabolic disease rate<br>Mid-treatment progressive metabolic disease rate<br>Death rate<br>Distant progression-free survival rate<br>Local progression-free survival rate

(Note) Not specified: indicate that the population may include diabetic or non-diabetic patients. NS: not specified. DM: diabetes mellitus.

Supplemental S4. The risk-of-bias assessment for randomised controlled trials

Author, year	Bias arising from the randomisation process	Bias due to deviations from intended intervention	Bias due to missing outcome data	Bias in the measurement of the outcome	Bias in the selection of the reported result	Overall
Chun, 2020 [20]	Some concerns	Low risk	Some concerns	Low risk	Low risk	Some concerns
Kim, 2021 [46]	Some concerns	Low risk	Some concerns	Low risk	Low risk	Some concerns
Skinner, 2021 [47]	Low risk	High risk	Some concerns	Low risk	Low risk	High risk
Tsakiridis, 2021 [21]	High risk	High risk	Low risk	Low risk	High risk	High risk
Tate, 2024 [48]	Some concerns	Low risk	Low risk	Low risk	Low risk	Some concerns

(Note) The risk-of-bias tool for randomised trials (RoB 2) was applied. Background colour caption: green: low risk of bias; yellow: some concerns; and red: high risk of bias.

Supplemental S5. The risk-of-bias assessment for the cohort studies

Author, year	Bias due to confounding	Bias due to the selection of participants	Bias in the classification of interventions	Bias due to deviations from intended interventions	Bias due to missing data	Bias in the measurement of outcomes	Bias in the selection of the reported result	Overall
Ferro, 2013 [32]	Moderate	Low	Low	Low	Low	Moderate	Serious	Serious
Skinner, 2013 [37]	Moderate	Low	Low	Low	Low	Low	Low	Moderate
Spratt, 2013 [38]	Moderate	Low	Low	Low	Low	Low	Serious	Serious
Taira, 2014 [41]	Moderate	Low	Low	Low	Low	Low	Serious	Serious
Adeberg, 2015 [11]	Moderate	Low	Moderate	Moderate	Moderate	Low	Serious	Serious
Ahmed, 2015 [19]	Moderate	Low	Low	Low	Low	Low	Low	Moderate
Jang, 2015 [33]	Moderate	Low	Low	Low	Low	Low	Serious	Serious
Van De Voorde, 2015 [44]	Moderate	Moderate	Low	Low	Low	Low	Low	Moderate
Spratt, 2016 [39]	Moderate	Low	Low	Low	Low	Low	Low	Moderate
Wink, 2016 [45]	Moderate	Low	Low	Low	Low	Low	Low	Moderate
Chang, 2017 [23]	Moderate	Low	Low	Low	Low	Moderate	Serious	Serious
Liu, 2017 [35]	Moderate	Low	Low	Low	Low	Low	Low	Moderate
Takiuchi, 2017 [42]	Moderate	Low	Low	Low	Low	Low	Serious	Serious
Li, 2019 [34]	Moderate	Low	Low	Low	Low	Low	Serious	Serious
Ranasinghe, 2019 [36]	Moderate	Low	Low	Low	Low	Low	Serious	Serious
Tsou, 2019 [43]	Moderate	Low	Low	Low	Low	Low	Low	Moderate
Yu, 2019 [22]	Moderate	Low	Low	Low	Low	Moderate	Low	Moderate
Cadeddu, 2020 [30]	Moderate	Low	Moderate	Low	Low	Low	Low	Moderate
Dağdelen, 2021 [31]	Moderate	Low	Low	Low	Low	Low	Low	Moderate
Stang, 2021 [40]	Moderate	Low	Low	Low	Low	Low	Serious	Serious

(Note) The risk of bias in non-randomised studies of interventions (ROBINS-I) tool was applied. Background colour caption: green; low risk of bias (the study is comparable to a well-performed randomised trial concerning this domain); yellow: moderate risk of bias (the study is sound for a non-randomised study concerning this domain but cannot be considered comparable to a well-performed randomised trial); orange: serious risk of bias (the study has some important problems); and red critical risk of bias (the study is too problematic to provide any useful evidence on the effects of intervention).

Supplemental S6. Other survival rate outcomes

Survival outcome	Study	Cancer	Comparison	Event rate	Odds ratio (95%CI)
**Group 1: metformin (NS) vs non-user (NS)**					
2-year local control rate	Jang (2015) [33]	Hepatocellular carcinoma	Metformin (NS) vs non-user (NS)	17/19 vs 44/57	2.51 (0.51, 12.32)
2-year intrahepatic metastasis-free survival rate	Jang (2015) [33]	Hepatocellular carcinoma	Metformin (NS) vs non-user (NS)	10/19 vs 17/57	2.61 (0.90, 7.58)
2-year extrahepatic metastasis-free survival rate	Jang (2015) [33]	Hepatocellular carcinoma	Metformin (NS) vs non-user (NS)	16/19 vs 36/57	3.11 (0.81, 11.95)
2-year survival rate after recurrence/progression	Takiuchi (2017) [42]	Cervical cancer	Metformin (NS) vs non-user (NS)	24/41 vs 221/437	1.38 (0.72, 2.64)
Biochemical failure-free survival rate	Cadeddu (2020) [30]	High-risk prostate cancer	Metformin (NS) vs non-user (NS)	54/70 vs 302/377	0.84 (0.45, 1.55)
Disease-specific survival rate	Cadeddu (2020) [30]	High-risk prostate cancer	Metformin (NS) vs non-user (NS)	68/70 vs 369/377	0.74 (0.15, 3.55)
Distant failure-free survival rate	Cadeddu (2020) [30]	High-risk prostate cancer	Metformin (NS) vs non-user (NS)	63/70 vs 334/377	1.16 (0.50, 2.69)
Biochemical recurrence-free survival rate	Dağdelen (2021) [31]	Prostate cancer	Metformin (NS) vs non-user (NS)	22/22 vs 64/72	5.50 (0.30, 99.75)
**Group2: metformin (DM+) vs non-user (NS)**					
Distant relapse rate	Adeberg (2015) [11]	Primary glioblastoma	Metformin (DM+) vs non-user (NS)	3/20 vs 67/256	0.5 (0.14, 1.75)
Multifocal relapse rate	Adeberg (2015) [11]	Primary glioblastoma	Metformin (DM+) vs non-user (NS)	3/20 vs 64/256	0.53 (0.15, 1.87)
2-year locoregional recurrence-free survival rate	Wink (2016) [45]	Locally advanced NSCLC	Metformin (DM+) vs non-user (NS)	42/59 vs 372/623	1.67 (0.93, 2.99)
Progression rate	Wink (2016) [45]	Locally advanced NSCLC	Metformin (DM+) vs non-user (NS)	23/59 vs 348/623	0.50 (0.29, 0.87)*
2-year recurrence-free survival rate	Chang (2017) [23]	Head and neck SCC	Metformin (DM+) vs non-user (NS)	27/39 vs 129/213	1.47 (0.70, 3.05)
**Group 3: metformin (DM+) vs non-user (DM+)**					
Cause-specific mortality rate	Taira (2014) [41]	Prostate cancer	Metformin (DM+) vs non-user (DM+)	3/126 vs 0/144	7.00 (0.35, 141.11)
Overall mortality rate	Taira (2014) [41]	Prostate cancer	Metformin (DM+) vs non-user (DM+)	47/126 vs 105/144	0.22 (0.13, 0.37)*
5-year local failure-free survival rate	Spratt (2016) [39]	Oropharyngeal cancer	Metformin (DM+) vs non-user (DM+)	96/102 vs 77/82	1.04 (0.31, 3.53)
5-year regional failure-free survival rate	Spratt (2016) [39]	Oropharyngeal cancer	Metformin (DM+) vs non-user (DM+)	95/102 vs 76/82	1.07 (0.35, 3.32)
Progression or mortality rate	Stang (2021) [40]	Locally advanced NSCLC	Metformin (DM+) vs non-user (DM+)	15/31 vs 3/11	2.50 (0.56, 11.23)
**Group 4: metformin (DM+) vs non-user (DM-)**					
Cause-specific mortality rate	Taira (2014) [41]	Prostate cancer	Metformin (DM+) vs non-user (DM-)	3/126 vs 30/2028	1.62 (0.49, 5.40)
Overall mortality rate	Taira (2014) [41]	Prostate cancer	Metformin (DM+) vs non-user (DM-)	47/126 vs 953/2028	0.67 (0.46, 0.97)*
5-year local failure-free survival rate	Spratt (2016) [39]	Oropharyngeal cancer	Metformin (DM+) vs non-user (DM-)	96/102 vs 1463/1561	1.07 (0.46, 2.51)
5-year regional failure-free survival rate	Spratt (2016) [39]	Oropharyngeal cancer	Metformin (DM+) vs non-user (DM-)	95/102 vs 1474/1561	0.80 (0.36, 1.78)
Progression or mortality rate	Stang (2021) [40]	Locally advanced NSCLC	Metformin (DM+) vs non-user (DM-)	15/31 vs 35/78	1.15 (0.50, 2.65)
**Group 5: metformin (DM-) vs non-user (DM-)**					
6-month progressive metabolic disease rate†	Chun (2020) [20]	Squamous or adenocarcinoma NSCLC	Metformin (DM-) vs non-user (DM-)	1/14 vs 0/1	0.15 (0.00, 8.05)
Mid-treatment progressive metabolic disease rate†	Chun (2020) [20]	Squamous or adenocarcinoma NSCLC	Metformin (DM-) vs non-user (DM-)	6/14 vs 0/1	1.50 (0.04, 52.54)
Death rate	Skinner (2021) [47]	Locally advanced NSCLC	Metformin (DM-) vs non-user (DM-)	24/86 vs 30/81	0.66 (0.34, 1.26)
Distant progression-free survival rate	Tsakiridis (2021) [21]	Locally advanced NSCLC	Metformin (DM-) vs non-user (DM-)	18/26 vs 12/28	3.00 (0.98, 9.19)
Local progression-free survival rate	Tsakiridis (2021) [21]	Locally advanced NSCLC	Metformin (DM-) vs non-user (DM-)	6/26 vs 4/28	1.80 (0.45, 7.28)

(Note) NS: The condition of diabetes mellitus was not specified. DM: diabetes mellitus. †Positron emission tomographic/computed tomographic scan response criteria in solid tumours-Progressive metabolic disease: an increase of ≥30% and an increase of at least 0.8 SUV units. NSCLC: non-small cell lung cancer. SCC: squamous cell carcinoma. *Significant difference.

Supplemental S7. Survival time outcomes

Study	Cancer	Comparison	No. of patients	Median survival time (months)	Median time differences between exposed and non-exposed groups (months)
**Overall survival time**					
Ahmed (2015) [19]	NSCLC	Metformin (DM+) vs non-user (DM+)	20 vs 20	14.3 vs 19.2	-4.9
Ahmed (2015) [19]	NSCLC	Metformin (DM+) vs non-user (DM-)	20 vs 126	14.3 vs 16.3	-2
Wink (2016) [45]	Locally advanced NSCLC	Metformin (DM+) vs non-user (NS)	59 vs 623	33 vs 23	10
Li (2019) [34]	Prostate cancer	Metformin (NS) vs non-user (NS)	16 vs 60	Mean: 82.8 vs 85.2	Mean difference: -2.4
Tate (2024) [48]	NSCLC	Metformin (DM-) vs non-user (DM-)	14 vs 1	59.64 vs Not reached	Not available
**Progression-free survival time**					
Adeberg (2015) [11]	Primary glioblastoma	Metformin (DM+) vs non-user (DM+)	20 vs 20	10.13 vs 4.67	5.46
Adeberg (2015) [11]	Primary glioblastoma	Metformin (DM+) vs non-user (DM-)	20 vs 236	10.13 vs 6.7	3.43
Ahmed (2015) [19]	NSCLC	Metformin (DM+) vs non-user (DM+)	20 vs 20	19.7 vs 10.1	9.6
Ahmed (2015) [19]	NSCLC	Metformin (DM+) vs non-user (DM-)	20 vs 126	9.7 vs 11.6	-1.9
Wink (2016) [45]	Locally advanced NSCLC	Metformin (DM+) vs non-user (NS)	59 vs 623	41 vs 15	26
Stang (2021) [40]	Locally advanced NSCLC	Metformin (NS) vs non-user (NS)	31 vs 89	36.4 vs 48.9	-12.5
Stang (2021) [40]	Locally advanced NSCLC	Metformin (DM+) vs non-user (DM-)	31 vs 78	36.4 vs 39.5	-3.1
Tate (2024) [48]	NSCLC	Metformin (DM-) vs non-user (DM-)	14 vs 1	55.8 vs Not reached	Not available
**Locoregional recurrence-free survival time**					
Ahmed (2015) [19]	NSCLC	Metformin (DM+) vs non-user (DM+)	20 vs 20	11.9 vs 15.5	-3.6
Ahmed (2015) [19]	NSCLC	Metformin (DM+) vs non-user (DM-)	20 vs 126	11.9 vs 14.1	-2.2
Wink (2016) [45]	Locally advanced NSCLC	Metformin (DM+) vs non-user (NS)	59 vs 623	Not reached vs 44	Not available
**Distant metastasis-free survival time**					
Ahmed (2015) [19]	NSCLC	Metformin (DM+) vs non-user (DM+)	20 vs 20	10 vs 17.4	-7.4
Ahmed (2015) [19]	NSCLC	Metformin (DM+) vs non-user (DM-)	20 vs 126	10 vs 13.4	-3.4
Wink (2016) [45]	Locally advanced NSCLC	Metformin (DM+) vs non-user (NS)	59 vs 623	Not reached vs 36	Not available

(Note) NSCLC: non-small cell lung cancer. DM: diabetes mellitus. NS: The condition of diabetes mellitus was not specified.
